# Weight Rich-Club Analysis in the White Matter Network of Late-Life Depression with Memory Deficits

**DOI:** 10.3389/fnagi.2017.00279

**Published:** 2017-08-23

**Authors:** Naikeng Mai, Xiaomei Zhong, Ben Chen, Qi Peng, Zhangying Wu, Weiru Zhang, Cong Ouyang, Yuping Ning

**Affiliations:** ^1^Department of Neurology, Southern Medical University Guangdong, China; ^2^Department of Neurology, The Affiliated Brain Hospital of Guangzhou Medical University (Guangzhou Huiai Hospital) Guangdong, China

**Keywords:** late-life depression, memory deficits, white matter, connectome, graph theory, rich-club

## Abstract

Patients with late-life depression (LLD) have a higher incident of developing dementia, especially individuals with memory deficits. However, little is known about the white matter characteristics of LLD with memory deficits (LLD-MD) in the human connectome, especially for the rich-club coefficient, which is an indicator that describes the organization pattern of hub in the network. To address this question, diffusion tensor imaging of 69 participants [15 LLD-MD patients; 24 patients with LLD with intact memory (LLD-IM); and 30 healthy controls (HC)] was applied to construct a brain network for each individual. A full-scale battery of neuropsychological tests were used for grouping, and evaluating executive function, processing speed and memory. Rich-club analysis and global network properties were utilized to describe the topological features in each group. Network-based statistics (NBS) were calculated to identify the impaired subnetwork in the LLD-MD group relative to that in the LLD-IM group. We found that compared with HC participants, patients with LLD (LLD-MD and LLD-IM) had relatively impaired rich-club organizations and rich-club connectivity. In addition, LLD-MD group exhibited lower feeder and local connective average strength than LLD-IM group. Furthermore, global network properties, such as the shortest path length, connective strength, efficiency and fault tolerant efficiency, were significantly decreased in the LLD-MD group relative to those in the LLD-IM and HC groups. According to NBS analysis, a subnetwork, including right cognitive control network (CCN) and corticostriatal circuits, were disrupted in LLD-MD patients. In conclusion, the disease effects of LLD were prevalent in rich-club organization. Feeder and local connections, especially in the subnetwork including right CCN and corticostriatal circuits, were further impaired in those with memory deficits. Global network properties were disrupted in LLD-MD patients relative to those in LLD-IM patients.

## Introduction

Late-life depression (LLD) is one of the most common psychological diseases in old age, with a prevalence that ranges from 3.5 to 7.5% (Weyerer et al., 2013). It is characterized by a heavy economic burden (Zivin et al., 2013) and is frequently accompanied by cognitive deficits (Butters et al., 2004) even after remission (Bhalla et al., 2006; Baba et al., 2010). In addition, numerous studies have shown that LLD patients have a higher risk of developing dementia, especially Alzheimers disease (AD), than healthy controls (Byers and Yaffe, 2011; Diniz et al., 2013; Heser et al., 2016). Among LLD, the patients with memory deficits are thought to have the most predictable signs of subsequent dementia (Rushing et al., 2014; Heser et al., 2016). However, little is known about the structural features of LLD with memory deficits (LLD-MD), which is crucial for understanding the disease process of LLD and the mechanism of AD development.

LLD is characterized by white matter lesions (Alexopoulos et al., 1997; Sneed et al., 2008), and these lesions are associated with some symptoms of LLD, such as cognitive deficits and affective symptoms (Kohler et al., 2010; Dalby et al., 2012). However, the distinctive white matter network topological structure underlying LLD, especially in individuals with memory deficits, is largely unknown. The connectome is an approach to help us understand the organization of the complex brain connective network through brain network construction and application of graph theory (Sporns et al., 2005). This technology has been applied to discover the underlying brain structural changes under neuropsychological diseases (Daianu et al., 2015; Gong and He, 2015; Schmidt et al., 2017). In LLD, the breakdown of global network properties and reductive connectivity between-hemisphere was found in previous studies of the white matter connective network (Bai et al., 2012; Li et al., 2017). But, there is still largely unknown about the topological organization, especially the role of hub, in LLD.

In the development of the connectome, several methods from graph theory have been used to describe the topological characteristics of the human brain network. Among them, the important role of the hub (the node with a relatively stronger connection in the network) has been identified, and the tendency to have more interconnection among hubs than random effects was called rich-club. Rich-club connectivity is important in information integration and its disruption is believed to be the cause of dysfunction in psychosis (Schmidt et al., 2017). Van den Heuvel and Sporns were the first to describe the existence of rich-club organization in the human brain network (van den Heuvel and Sporns, 2011). Since then, many studies have started to apply rich-club analysis to describe network properties in disease states (van den Heuvel et al., 2013; Collin et al., 2014; Daianu et al., 2015; Schmidt et al., 2017), and their contributions have help us to obtain the better understanding of human brain network topology, especially under the disease states of neuropsychological disorders. According to van den Heuvel et al. rich-club is crucial in information integration among different functional modules (van den Heuvel et al., 2013), while functional modules disruption was found in major depressive disorder and correlated with the feelings of helplessness (Peng et al., 2014), indicated that rich-club organization may be relevant to depression. What is more, impaired rich-club coefficient was also found on schizophrenia, which was characterized by psychiatric symptoms (van den Heuvel et al., 2013). However, direct evidence from LLD was lacked. AD, which is well known for memory deficits and can be the result of LLD-MD, was found relatively preserved rich-club organization, while cognitive function was consistent with non-rich-club connectivity (Daianu et al., 2015). According to the evidences as described above, we hypothesized that as for LLD-MD, a neuropsychological disease with depression symptom, memory deficits and high risk of developing AD, may be characterized by both deficits rich-club organization and disrupt non-rich-club connectivity. So we designed this study to verify this hypothesis.

What is more, in order to obtain a more comprehensive understanding of LLD-MD, global network properties and Network-based statistics (NBS) also applied in the current study. Global network properties, such as clustering coefficient, the shortest path length, connective strength, efficiency and others in the global network level, are used to measure the efficiency of a network's information processing. NBS is a powerful tool to identify the different subnetworks between groups with relatively weaker control of the family-wise error rate (FWER). Numerous studies have applied NBS to distinguish the experimental effects on the brain network (Cocchi et al., 2014; Myung et al., 2016). We expect that the application of NBS will allow us to identify the effects of memory deficits in LLD's structural network. Overall, the motivation of this study is to determine the unique structural changes in LLD-MD patients as compared to LLD patients with intact memory (LLD-IM) and healthy controls (HC) through connectome.

To the best of our knowledge, this is the first time to identify the characteristics of LLD through the rich-club coefficient. We used probabilistic tractography to construct white matter networks of diffusion tensor imaging (DTI) in 15 LLD-MD patients, 24 LLD-MI patients and 30 HC. Nodes were defined by registering automatic anatomical labeling (AAL) to DTI through a T1 structural image. Weight rich-club analysis, global networks properties and NBS analysis were applied to describe the topological features of LLD-MD and determined whether rich-club organization and non-rich-club connectivity were disrupted in LLD-MD. Furthermore, the correlation between cognitive function and white matter network topological features was investigated.

## Materials and methods

### Participants

Thirty-nine LLD patients were recruited in The Affiliated Brain Hospital of Guangzhou Medical University and 30 HC individuals in a local community from June, 2014 to June, 2016. All of them were of Han Chinese ethnicity and were right-handed. Written informed consent was obtained from all of the participants. Research ethics permission was approved by The Affiliated Brain Hospital of Guangzhou Medical University ethics committee.

LLD patients met the following inclusion criteria: (1) age >55 years old; (2) met the Diagnostic and Statistical Manual of Mental Disorders, Fourth Edition (DSM-IV) criteria for major depression disorder after the age of 55; (3) diagnosis confirmed by trained psychiatrists from the Affiliated Brain Hospital of Guangzhou Medical University; (4) able to cooperate in neuropsychological tests; and (5) clinical stage confirmed by their psychiatrists not to be in a relapse stage. Exclusion criteria included: (1) inability to cooperate with neuropsychological tests; (2) a history of the other major psychiatric disorders, such as bipolar affective disorder and schizophrenia; (3) a family history of bipolar disorder and/or schizophrenia; (4) a primary neurological illness, such as stroke and brain tumor; and (5) a physical disease that can cause emotional problems, such as hypothyroidism and anemia.

We further divided LLD patients into two groups: patients with memory deficits and patients with intact memory. For the Auditory Verbal Learning Test-delayed recall score (AVLT-N5), patients with age- and education-adjusted scores ≤1.5 SD (the cutoff was AVLT-N5 ≤4 for ages 50–59 years) were identified as having memory deficits. The rest of the patients were defined as having intact memory. Of the 39 LLD patients, 15 of them were classified as LLD-MD patients, and 24 as LLD-IM patients.

Considering that mild cognitive deficit was common in LLD, which is caused by disease effects, all HC were required to have Mini-Mental State Examination (MMSE) scores ≥24 and age- and education-adjustedAVLT-N5 scores >1.5 SD, and those who had been diagnosed with major depressive disorder before the study were excluded. Other exclusion criteria were similar to those for the LLD groups.

### Neuropsychological tests

A full-scale battery of neuropsychological tests was assessed prior to MRI scans. The battery included the MMSE, the Hamilton Rating Scale for Depression (HRSD), the AVLT, the Trail-Making Test (TMT), the Stroop Color and Word Test (Stroop), the Digit Span Test (DST), the Symbol-Digit Modality Test (SDMT), the Logical Memory Test (LMT) and the participants medical records. One of the subjects in the LLD-MD group did not complete the TMT.

We separated cognitive function into 3 domains: executive function, processing speed and memory. Each of the domains was calculated by the battery of neuropsychological tests described above. The calculations followed the procedure described by Sheline et al. (2006). In brief, the measure of executive function was calculated by average Z scores of (1) the reverse of the difference between completion time of TMT Part B and TMT Part A; (2) the reverse of the difference between completion time of Stroop Part C and Stroop Part A; and (3) the difference between the DST backward scores and the DST forward scores. Processing speed was calculated by the average Z scores of (1) the reverse completion time of Stroop Part A; (2) the reverse completion time of TMT Part A; and (3) the correct number of SDMT. Memory was calculated by the average Z scores of (1) the average scores of AVLT from N1 to N5; and (2) the long-term scores of LMT.

### MRI acquisition

Participants were scanned using a 3.0-Tesla Philips Achieva scanner (Phlips, Best, The Netherlands) at The Affiliated Brain Hospital of Guangzhou Medical University. Before scanning, a T2-weight image was applied to rule out cerebral infarction, tumors, and major white matter lesions. The DTI scanning parameters were as follows: direction = 32, b_0_ = 1,000 s/mm^2^, repetition time (TR) = 10,015 ms, echo time (TE) = 92 ms, flip angle = 90°, imaging matrix = 128^*^128 mm^2^, FOV = 256^*^256 mm^2^, 75 contiguous slices, and voxel dimension of 2^*^2^*^2 mm^3^. High-resolution T1-weight images were acquired from a 3D spoiled gradient echo sequence: *TR* = 8.2 ms, *TE* = 3.8 ms, slices thickness = 1 mm, and FOV = 256^*^256 mm^2^ (matrix = 256^*^256^*^188).

### Data preprocessing

Data preprocessing was performed using FMRIB's Diffusion Toolbox (FMRIB's Software Library, FSL) for the following procedures. First, the eddy current correlation was used to correct the distortions from stretches and shears in the DTI as well as correct for simple head motion. Second, b_0_ image extraction and brain extraction (fractional intensity threshold = 0.2) were performed. Third, Bedpostx (Bayesian Estimation of Diffusion Parameters Obtained using Sampling Techniques) was used to set up the distribution of fiber orientation at each voxel. In T1 images, BET was utilized for brain extraction (fractional intensity threshold = 0.3).

### Network construction

The brain network contains nodes and edges. To determine the nodes in the network, we selected 90 areas of gray matter regions of the cerebrum from AAL, which included 45 regions of cortical and subcortical structures in each hemisphere. Edge definition was accomplished using the connectivity probability between each pair of nodes in the network. Network construction was performed by PANDA (Cui et al., 2013). The details of network construction were as follows.

#### Node definition

The procedure was completed following Gong's description (Gong et al., 2009). Briefly, T1 images were non-linearly co-registered to the MNI152_T1_Template. The inverse warp was obtained from a previous step, and the transformation matrix from T1 to b0 were combined to warp the AAL regional mask from the MNI space to the individual T1 space and b_0_ space successively. Entirely, 90 AAL regions were executed using the same procedures as described above to establish the seed mask. For each of the seed masks, the rest 89 regional masks were merged as the terminal mask.

#### Edge definition

As we described above, probabilistic tractography (FSL 5.09) was used to define the edge of the brain network. For each voxel in the seed mask, 5,000 streamlines were sampled. Depending on the distribution of the orientation set up by Bedpostx, each fiber was drawn. The tract was established every 0.5 mm to the other 89 masks and terminated in a terminal mask to prevent tracking in the loop. Finally, connectivity probabilities from the seed mask to the rest of the 89 target masks were established. The probability was defined as weight. After 90 seed masks were performed, the same procedure described above was carried out and a 90^*^90 connective matrix was constructed for each subject.

It is impossible to determine the direction between node i and node j by probabilistic tractography. Therefore, we defined the undirectional connective matrix using Schmidt's description (Schmidt et al., 2017), and the probabilities of node i and node j were calculated by the average of weight ij and weight ji.

### Network analysis

In the current study, we attempt to characterize the differences in the white matter network between LLD-MD and LLD-IM patients by network analysis, which contained 3 parts, including weighted rich-club analysis, global efficiency assessment and network-based statistics.

#### Weighted rich-club analysis

Rich-club organization is to describe the tendency connection among a set of the most important nodes in the network and is usually represented by the rich-club coefficient. To investigate whether the rich-club organization was affected in 39 LLD patients or just in 15 LLD-MD patients among them, we conducted weight rich-club analysis. Although probabilistic tractography can reflect the real white matter connection between brain region, but its node's strength show no bias in different nodes and it is not available to figure out the most importance nodes in the network, which is the first step of weight rich-club analysis. Therefore, the network matrix should be normalized before weight rich-club analysis to distinguish the important nodes from the others. The weight of the connection is influenced by the voxels size of both the seed region and the target region of the network, which constructed by probabilistic tractography. In our study of probabilistic tracking, 5,000 streamlines were generated pre-voxel in the seed mask and ended at the target mask, which means that a larger seed mask and target mask will lead to more streamlines. The definition of weighted rich-club node is depended on the relative connective strength from the network. Furthermore, volume differences become larger in older subjects, and the gray matter voxels should also take into account. Thus, we normalized the network matrix with the following equation:

$$\begin{array}{l} normalized\text{\_}W_{ij}=\frac{W_{ij}}{\begin{array}{c} \left(seed\;voxels/gray\;matter\;voxels\right)\times \\ \;\left(target\;voxels/gray\;matter\;voxels\right) \end{array}}, \end{array}$$

where *W*_*ij*_ is the probability connective weight between a pair of nodes (*i* and *j*) in the network. With this equation, we ruled out the effects of seed voxels, target voxels and whole brain gray matter volumes on the probabilistic tractography in the weighted rich-club analysis to display the relative strongest connection in the network. And the procedure of normalizing was only taken in weight rich-club coefficient calculation and rich-club node definition to avoid that those over strong central connections would mask the effect of other non-central connections in network properties' calculation. Before normalized the network matrix, the edges that existed in less than 20% of the subjects in the group were excluded from the network.

According to Schmidt and Opsahl's description (Opsahl et al., 2008; Schmidt et al., 2017), the weighted rich-club analysis was performed with the following procedures. First, richness factor r is defined as the faction of the strongest nodes among the network (here we defined r as the rank from 14/15 to 1/15, which ensured that the rich-club node number is an integer), and E > r is defined as the edges in the rich-club subnetwork with richness factor >r. In a particular richness factor r, a rich-club subnetwork was constructed by the interconnection among the top r^*^90 strongest nodes, and the weight rich-club coefficient φ^ω^(*r*) can be calculated by the following equation: $\varphi^{\omega }\left(r\right)=\omega_{>r}/\overset{{\text{E}}_{>r}}{\underset{l=1}{\sum }}\omega_{\text{l}}^{\text{rank}}$, where ω_>*r*_ is the weight of the rich-club network and $\overset{{\text{E}}_{>r}}{\underset{l=1}{\sum }}\omega_{\text{l}}^{\text{rank}}$ is the sum of the strongest E_>*r*_ connections in the whole network. Second, to eliminate the random effect, 1,000 random networks were constructed by a weight reshuffle according to Opsahl's description (Opsahl et al., 2008). Then, a normalized rich-club coefficient ($\varphi_{\text{norm}}^{\omega }\left(r\right)$) was determined by the ratio of $\varphi^{\omega }\left(r\right)/\varphi_{\text{random}}^{\omega }\left(r\right)$, where $\varphi_{\text{random}}^{\omega }\left(r\right)$ is the average of 1,000 random networks' rich-club coefficients. $\varphi_{\text{norm}}^{\omega }\left(r\right)$ >1 was considered to indicate the existence of rich-club organization for the network. Finally, we constructed the distribution of the rich-club coefficient in a series of r levels in LLD-MD and LLD-IM patients and HC individuals.

After constructing the rich-club subnetwork, nodes besides the rich-club was defined as a local region. The feeder connections was the connection between rich-club regions and local regions, and the local connections was represented as the interconnection among local regions (shown in **Figure 2**). The connective average strength of rich-club connections, feeder connections and local connections were divided the number of connections of each subnetwork from their total network's weight.

#### Global network properties

To describe the white matter network topology in global level, we calculated several global network properties: clustering coefficient (Cp), shortest path length (Lp), efficiency (E_glob), connective strength (S), fault tolerant efficiency (Eloc) and assortativity (r) (Newman, 2002; Leung and Chau, 2007). All global network properties were calculated with the GRETNA toolkit (Wang et al., 2015) or home make matlab program. To rule out the spurious connections, if the connection existed in less than 20% of the group subjects, it was excluded from the connective matrix before calculating the global network properties.

##### Global clustering coefficient

The global clustering coefficients defined as the average of the likelihood of a neighbor to neighbor connection. The greater value is represented to a larger extent of the local interconnectivity of a network. For a network G, the equation is $\text{global}\;{\text{C}}_{\text{p}}=(1/\text{N})\times \Sigma \{\sum_{\text{i}\epsilon \text{G}}[2/{\text{k}}_{\text{i}}({\text{k}}_{\text{i}}-1)\sum_{\text{j},\text{k}}{\left(\omega_{\text{ij}}\omega_{\text{jk}}\omega_{\text{ki}}\right)}^{\frac{1}{3}}]\}$, in which k_*i*_ is the degree of node i and ω_ij_ is the weight between node i and node j. *N* = 90.

##### Global shortest path length

The global shortest path length is defined as the average of all shortest lengths between each pair of nodes in the network. The smaller value is represented for the faster transfer speed of information in the brain. For a network G, the equation is Global L_p_ = [1/N(N − 1)] × Σ_i≠jϵG_L_ij_], where L_ij_ is the shortest path length between node i and node j and *N* = 90.

##### Small world properties

Before small world property calculations, 1,000 random networks were generated that maintain the same nodes and edges as the original network but also maintain differences in distribution. The small world properties contained the normalized global shortest path length (λ, $\text{λ}=\text{Global }{\text{L}}_{\text{p}}^{\text{real}}/\text{Global }{\text{L}}_{\text{p}}^{\text{rand}}$), normalized global clustering coefficient (γ, $\text{γ}=\text{Global }{\text{C}}_{\text{p}}^{\text{real}}/\text{Global }{\text{C}}_{\text{p}}^{\text{rand}}$) and small-worldness (σ, σ = γ/λ), while $\text{Global}\;{\text{L}}_{\text{p}}^{\text{rand}}$ and $\text{Global }{\text{C}}_{\text{p}}^{\text{rand}}$ are the means of 1,000 random network global clustering coefficients and the global shortest path length, respectively, λ ≈ 1, γ > 1, and σ > 1 indicate the existence of small world properties.

##### Network connective strength

The connective strength of node i is defined as the sum of the connective weight, which directly connects to the node i. Network connective strength is the average of all of the nodal connective strength in the network. For a network G, the equation is $S=\;(1/\text{N})\times \Sigma [\sum_{\text{i}\in \text{G}}(\sum_{\text{j}\in \text{G}}\omega_{\text{ij}})$], where ω_ij_ is the weight between node i and node j. The greater network connective strength is represented by the greater connection connective strength in the network.

##### Global efficiency

The global efficiency is represented as the information transfer efficiency of the network. For a network G, the equation is ${\text{E}}_{\text{glob}}=[1/\text{N}(\text{N}-\text{1})]\times \sum_{\text{i}\neq \text{j}\in \text{G}}1/{\text{L}}_{\text{ij}}$, where L_ij_ is the shortest path length between node i and node j.

##### Global fault tolerant efficiency

The Eloc of node i is defined as how much of the network is fault tolerant when the first neighbors of node i were removed from the network. The global Eloc is the average of nodal Eloc in the network. The greater global Elocis represented for the greater fault tolerance of the network. For a network G, the equation is ${\text{E}}_{\text{loc}}=(1/\text{N})\times \sum_{\text{i}\in \text{G}}{\text{E}}_{\text{glob}}({\text{G}}_{\text{i}})$.

##### Assortativity

Assortativity is defined as one node that tends to connect with other similar nodes in the network. In this study, we calculate assortativity with Pearson correlation coefficients of connective strength between each pair of linked nodes in the network as Leung and Chau (2007). The equation is ${\text{r}}^{\omega }=\frac{{\text{H}}^{-1}\sum_{\varphi }\left(\omega_{\varphi }\prod_{\text{i}\in \text{F}\left(\text{φ}\right)}{\text{k}}_{\text{i}}\right)-{\left[\frac{{\text{H}}^{-1}}{2}\sum_{\varphi }\left(\omega_{\varphi }\sum_{\text{i}\in \text{F}\left(\text{φ}\right)}{\text{k}}_{\text{i}}\right)\right]}^{2}}{\frac{{\text{H}}^{-1}}{2}\sum_{\varphi }\left(\omega_{\varphi }\sum_{\text{i}\in \text{F}\left(\text{φ}\right)}{\text{k}}_{i}^{2}\right)-{\left[\frac{{\text{H}}^{-1}}{2}\sum_{\varphi }\left(\omega_{\varphi }\sum_{i\in F\left(\varphi \right)}{\text{k}}_{\text{i}}\right)\right]}^{2}}$, where the edge of the network is sorted by ascending values, H is the total weight of all the edges in the network, ω_φ_ is the weight of the φ th edge, and *F* (φ) is the pair of nodes connected by the φ th edge. If M is represented as the total degree of the network, then φ = 1 ~ M.

##### Density of network

Density of network is the fraction of present connections to possible connections. In this study the possible connections is equal to N(N − 1)/2.

#### Network-based statistics (NBS)

To further determine the different connections between a pair of LLD groups. NBS was performed following Zalesky's methods (Zalesky et al., 2010) with NBS connectome software (http://www.nitrc.org/projects/nbs/). Before NBS, a binary mask that contains 80% of the edges for either the LLD-MD group or the LLD-IM group was applied to the matrix of the LLD groups.

### Statistical analysis

The group differences in age, years of education, HRDS, MMSE, and AVLT-N5 were determined by one-way ANOVA test. The gender data was analyzed by chi-square test.

Group differences in the normalized rich-club coefficient were calculated by permutation test (10,000 permutations) and adjusted for age, education, and gender. Findings were corrected for the levels of rich-club factors (*P* < 0.05/14). To further test the group differences in different rich-club factor levels, the area under curve of rich-club coefficients in different rich-club factor levels was calculated, and permutation test (10,000 permutations) was used to determine the group differences (*P* < 0.05). Rich-club regions were determined by the procedure Schmit described (Schmidt et al., 2017). For sorting by node connective strength, we selected the 12 most powerful nodes (2/15^*^90nodes, ≈13.3% of the whole network nodes) from HC and defined them as the rich-club region. The decision was made from the results of the permutation test, which indicated significant differences among three groups appeared in the *r* = 2/15 level and matched the values described in a previous article (van den Heuvel and Sporns, 2011; Schmidt et al., 2017) in which rich-club was selected from the top 15 or 14.6% nodes based on their node connective strength or degree. In the rich-club region, edges that existed in more than the 80% of the subjects were selected as the rich-club connections.

To determine the group differences in cognitive function and global network properties, comparisons were made using ANCOVA to remove the effects of age, years of education and gender. *Post hoc* analysis was performed using LSD method in all of the above analyses. *P* < 0.05 was considered to be statistically significant. The potential relationships between cognitive function (executive function, processing speed and memory) and global network properties in all participants were tested using a Pearson correlation analysis and adjusted with HDRS. The findings were corrected for the number of performed tests in each network property (*P* < 0.05/3).

To determine the significant differences in the subnetwork connection between the LLD-MD and LLD-IM groups. NBS was taken and the procedure developed by Zalesky and Li was used (Zalesky et al., 2010; Li et al., 2017). First, the *t*-test statistic threshold was chosen by primary threshold (*P* < 0.01). Then, a two sample one-tail *t*-test (LLD-MD < LLD-IM) was computed for difference edges between LLD-MD and LLD-IM. A set of suprathreshold links were constructed according to the statistic threshold. Then, a connected graph component was determined by breadth search, and the component size M was established by the sum of test statistics values across all connections in the component. A permutation test (5,000 permutations) was used to correct for multiple comparisons. The size of the largest component was recorded in each permutation and generated a random component size distribution. Finally, the correct *P* value was determined by the position of the M in the random component size distribution. The significantly different subnetwork between the pair of LLD groups was obtained. Connective strength of the NBS subnetwork was calculated.

To further explore the relationship between cognitive function and regional network properties (i.e., rich-club connective average strength, feeder connective average strength, local connective average strength and NBS subnetwork connective strength), a Pearson correlation test was applied to the LLD groups and the HC group and adjusted with HDRS. The findings were corrected for the number of tests performed for each network property (*P* < 0.05/3). Further, the relation between the subnetworks (rich-club, feeder, and local) connective average strength and cognitive function (executive function, processing speed and memory) will use stepwise multiple regression to confirm. Cognitive function will set as the dependent variable and subnetworks (rich-club, feeder, and local) connective average strength will set as the independent variable. HDRS will enter the regression equation as a covariance.

The relation between HDRS and rich-club coefficient and subnetwork connective strength were performed with Pearson correlation test.

## Results

### Demographics and neuropsychiatric results

The demographic data is shown in Table 1. There were no significant differences in age, education and gender in the three groups (all *P* > 0.1). Among the LLD-MD group, 3 patients received a serotonin noradrenaline re-uptake inhibitor (SNRI), 10 patients received selective serotonin reuptake inhibitors (SSRI), 1 patient received tricyclic antidepressants (TCAs), 1 patient received noradrenergic and specific serotonergic antidepressants (NaSSA), and 5 patients received benzodiazepines (BZ) as a combination treatment within the last 3 months. Among the LLD-IM group, 4 patients received SNRI, 11 received SSRI, 1 received TCAs, 2 received NaSSA, 6 did not take any antidepressant medicine and 13 patients received BZ as a monotherapy or combination treatment within the last 3 months. No difference was found between the LLD-MD and LLD-IM groups between antidepressant treatment (χ^2^ = 4.697; *P* = 0.320) and BZ (χ^2^ = 1.612; *P* = 0.204). Differences were found in the HRSD, AVLT-N5, and MMSE (*P* < 0.05). After adjusted with age, education and gender, executive function, processing speed and memory were still different among the three groups (all *P* < 0.05).

**Table 1:** Demographics and neuropsychiatric results in the LLD with memory deficits and LLD with intact memory groups as well as the healthy control group.

	**LLD-MD (*n* = 15)**	**LLD-IM (*n* = 24)**	**HC (*n* = 30)**	***P*-values**
	**Mean (SD)**	**Mean (SD)**	**Mean (SD)**	
Age	64.47 ± 6.87	66.21 ± 5.57	66.23 ± 4.95	0.567
Education	8.53 ± 3.76	9.71 ± 3.82	10.65 ± 3.00	0.158
Gender	3M/12F	6M/18F	6M/24F	0.891
HRSD	9.13 ± 7.92^a^	10.79 ± 7.11^a^	1.27 ± 3.04	<0.0001^*^
AVLT-N5	0.80 ± 1.15^a^^b^	6.71 ± 2.16	7.30 ± 1.99	<0.001^*^
MMSE	21.40 ± 4.45^a^^b^	26.54 ± 2.40	27.37 ± 1.75	<0.001^*^
**COGNITIVE DOMAINS**				
Executive function	−0.375 ± 0.332^a^^b^	0.065 ± 0.635	0.139 ± 0.462	0.003^#^
Processing speed	−0.791 ± 0.721^a^^b^	−0.034 ± 0.692^a^	0.423 ± 0.710	<0.001^#^
Memory	−1.140 ± 0.362^a^^b^	0.197 ± 0.700	0.413 ± 0.714	<0.001^#^

* Significant according to one-way ANOVA (P < 0.05);

# *Significant according to one-way ANCOVA (adjusted for age, education and gender, P < 0.05)*.

a *Significant according to LSD post hoc comparisons (vs. HC; P < 0.05)*.

b *Significant according to LSD post hoc comparisons (vs. LLD-IM; P < 0.05)*.

*Group differences in all variables were calculated according to ANOVA, except for gender, which was determined through a chi-square test; LLD-MD, late-life depression with memory deficits; LLD-IM, late-life depression with intact memory; HC, healthy controls*.

Furthermore, both the LLD-MD and LLD-IM groups had significant decreases in the HRSD scores compared to the HC group (all *P* < 0.05). No differences were found between the LLD-MD and LLD-IM groups (*P* = 0.397) in HRSD. No correlation was found between HRSD and cognitive function in the LLD groups (all *P* > 0.1; show in Supplemental Table 2). LLD-MD patients had lower AVLT-N5 and MMSE scores than those of LLD-IM patients and HC individuals (all *P* < 0.05). The scores of executive function, processing speed and memory were significantly lower in LLD-MD patients than those in LLD-IM patients and HC individuals (all *P* < 0.05). No significant difference was found in executive function and memory between LLD-IM patients and HC individuals (all *P* > 0.05), while processing speed was significant lower in the LLD-IM group than that in the HC group (*P* = 0.042).

### Weight rich-club analysis

**Figure 1:**
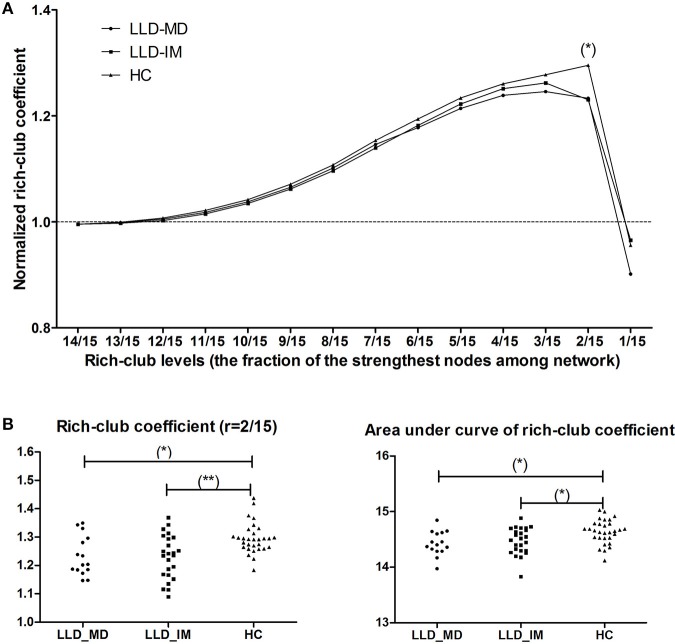
**(A)** Normalized rich-club coefficient at different rich-club levels, which are defined as a fraction of the most powerful node connective strength in the LLD-MD (*n* = 15), LLD-IM (*n* = 24), and HC (*n* = 30) groups. (^*^) significantly lower normalized rich-club coefficient in LLD patients than HC individuals, *P* < 0.05/14. **(B)** Group differences in rich-club coefficient (*r* = 2/15) and the area under the curve of the rich-club coefficient among the 3 groups (^*^P < 0.05; ^**^P < 0.05/14).

Under a range of rich-club levels (from *r* = 12/15 to 2/15), normalized rich-club coefficient was detected in 3 groups, for which rich-club organization was defined as the network connection with a normalized rich-club coefficient >1. We found intergroup differences in *r* = 2/15 through permutation testing (*P* = 0.001249, 10,000 samples, pass FDR correction for multiple comparisons, as shown in Figure 1). Indicated by LSD *post hoc* comparisons, the normalized rich-club coefficient was decreased in the LLD-MD and LLD-IM groups compared with that in the HC group (*P* = 0.005; *P* = 0.001). No difference was found between the LLD-MD and LLD-IM groups (*P* = 0.998; shown in Figure 1). The area under the curve of the rich-club coefficient was significantly different among groups (*P* = 0.008438), and compared with HC group, LLD-MD and LLD-IM groups was showed significant decreased (*P* = 0.006; *P* = 0.015). No difference was found between the LLD groups (LLD-MD and LLD-IM) (*P* = 0.491) on the area under the curve of the rich-club coefficient (shown in Figure 1).

**Figure 2:**
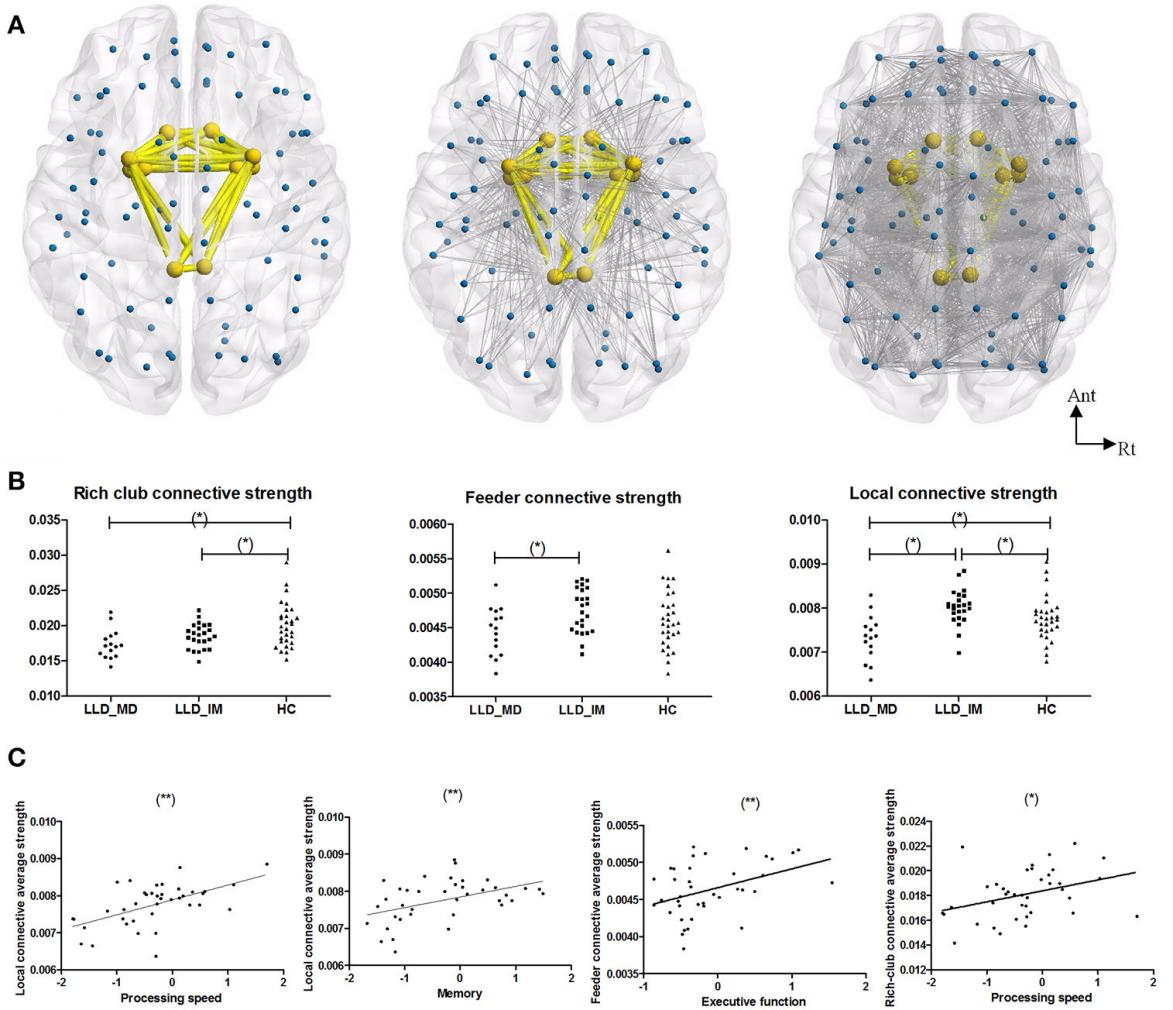
**(A)** From left to right, the images represent the rich-club connection, feeder connection and local connection. Rich-club included bilateral olfactory, cingulum_post, amygdala, putamen, and pallidum. The rich-club connections are colored in yellow, and the feeder and local connections are colored in gray. **(B)** From left to right, the graphs represent group differences in rich-club connective average connective strength, feeder connective average connective strength, and local connective average connective strength in a normalized weight probability connective network among 3 groups (^*^P < 0.05 in LSD *post hoc* comparisons). **(C)** A Pearson correlation between subnetwork average connective strength and cognitive function in LLD groups (LLD-MD+LLD-IM) (adjust with HDRS; ^*^P < 0.05; ^**^P < 0.05/3). The nodes and connections were mapped using BrainNet Viewer software (http://www.nitrc.org/projects/bnv/).

The rich-club regions were defined according to the description in the statistical analysis section, and the rich-club regions comprised 10 nodes, including bilateral olfactory, cingulum_post, amygdala, putamen and pallidum. Feeder connections (non-rich-club regions connected to rich-club regions) and local connections (interconnections among non-rich-club regions) were constructed based on rich-club regions (shown in Figure 2).

A difference was found in the rich-club, feeder and local connective average strength among the three groups (*P* = 0.003630, 0.049758, & 0.000224 respectively, permutation testing 10,000 samples). The rich-club connective average strengths of the LLD-MD and LLD-IM group were significantly weaker than the HC groups (*P* = 0.001; *P* = 0.035); the feeder connective average strengths of the LLD-MD group were significantly weaker than the LLD-IM groups (*P* = 0.015); the local connective average strengths of the LLD-MD group were significantly weaker than the LLD-IM and HC groups (*P* < 0.001; *P* = 0.011), while the LLD-IM group had a higher value than the HC group in local connective average strengths (*P* = 0.032; shown in Figure 2).

In LLD patients, the local connective average strength was positively correlated with processing speed (*r* = 0.555, *P* < 0.001) and memory (*r* = 0.432, *P* = 0.004), and the tendency was correlated with executive function [*r* = 0.361, *P* = 0.026 (ϵ(0.05/3,0.05))]; the feeder connective average strength was positively correlated with executive function (*r* = 0.416, *P* = 0.009) and processing speed (*r* = 0.411, *P* = 0.010); the rich-club connective average strength was tendency positively correlated with processing speed (*r* = 0.369, *P* = 0.023) and memory (*r* = 0.330, *P* = 0.043) (partly shown in Figure 2 according to the finding of stepwise multiple regression). No correlation between cognitive function and subnetwork's connective strength was found in HC individuals (details are shown in Supplemental Table 2).

Indicated by stepwise multiple regression, after adjusted of HDRS, feeder connective average strength (beta = 0.417; *P* = 0.009) was acted as the influence factor of executive function; local connective average strength (beta = 0.520; *P* = 0.001) and rich-club connective average strength (beta = 0.281; *P* = 0.043) were acted as the influence factor of processing speed; local connective average strength (beta = 0.477; *P* = 0.004) was acted as the influence factor of memory.

HDRS show no correlation with rich-club coefficient and subnetwork connective strength in LLD and HC groups respectively (all *P* > 0.05).

### Network property

Small world and global topology of the white matter network is shown in Table 2.

**Table 2:** Comparison of small world and global topology among LLD patients with memory deficits and LLD patients with intact memory as well as HC.

	**LLD-MD (*n* = 15)**	**LLD-IM (*n* = 24)**	**HC (*n* = 30)**	***P*-values**
	**Mean (SD)**	**Mean (SD)**	**Mean (SD)**	
γ	1.75 ± 0.107	1.76 ± 0.082	1.74 ± 0.108	0.852
λ	1.23 ± 0.017	1.23 ± 0.011	1.23 ± 0.012	0.680
σ	1.42 ± 0.072	1.43 ± 0.064	1.42 ± 0.077	0.832
Cp	3.82E−03 ± 5.14E−04	3.83E−03 ± 4.15E−04	3.69E−03 ± 5.43E−04	0.519
Lp	48.25 ± 3.75^a^^b^	44.85 ± 2.55	45.79 ± 3.13	0.006^*^
S	0.393 ± 0.030^a^^b^	0.422 ± 0.023	0.417 ± 0.029	0.008^*^
E_glob	2.08E−02 ± 1.62E−03^a^^b^	2.24E−02 ± 1.19E−03	2.19E−02 ± 1.49E−03	0.008^*^
Eloc	2.10E−02 ± 1.57E−03^a^^b^	2.26E−02 ± 1.21E−03	2.23E−02 ± 1.53E−03	0.008^*^
r	0.278 ± 0.040^a^	0.276 ± 0.027^a^	0.303 ± 0.027	0.006^*^
Density	0.642 ± 0.016^b^	0.633 ± 0.012	0.644 ± 0.016^b^	0.028^*^

* *Significant according to an one-way ANCOVA (adjusted for age, education and gender, P < 0.05)*.

a *P-value < 0.05 in LSD post hoc comparisons (vs. HC)*.

b *P-value < 0.05 in LSD post hoc comparisons (vs. LLD with intact memory)*.

*Cp, clustering coefficient; Lp, shortest path length; S, connective strength; E_glob, efficiency; Eloc, fault tolerant efficiency; r, assortativity; Density, density of network*.

#### Small world properties

The white matter network showed small world organization in three groups, which was defined as γ > 1, λ ≈ 1, and σ(γ/λ) > 1. No significant difference was found in the small world properties among the three groups (all *P* > 0.1).

#### Global topology

None of the individual network showed disconnected network. Intergroup differences were found in Lp, E_glob, S, Eloc, r and Density of network (all *P* < 0.05). In addition, lower E_glob, S and Eloc was found in the LLD-MD group compared to those in the other groups (all *P* < 0.05). Compared to LLD-IM and HC groups, LLD-MD group had increased Lp (*P* = 0.002; *P* = 0.03). A lower r was found in the LLD-MD and LLD-IM groups than the HC group (*P* = 0.02; *P* = 0.003). LLD-IM displayed lower density of network than LLD-MD and HC groups (*P* = 0.050; *P* = 0.012). Furthermore, after adjusted with HDRS, S, Eloc and r were positively correlated with processing speed (all *P* < 0.05/3) across all participants, while E_glob was tendency positive correlated with processing speed [all *P*ϵ(0.05/3,0.05)]. S, E_glob and Eloc were positively correlated with memory (all *P* < 0.05/3), while r had a positive tendency correlated with memory [*P*ϵ(0.05/3,0.05)]. Lp had a negative tendency correlated with processing speed (*P* < 0.05), and a negative correlated with memory (*P* < 0.05/3). Details are shown in Supplemental Table 1.

### NBS analysis

**Figure 3:**
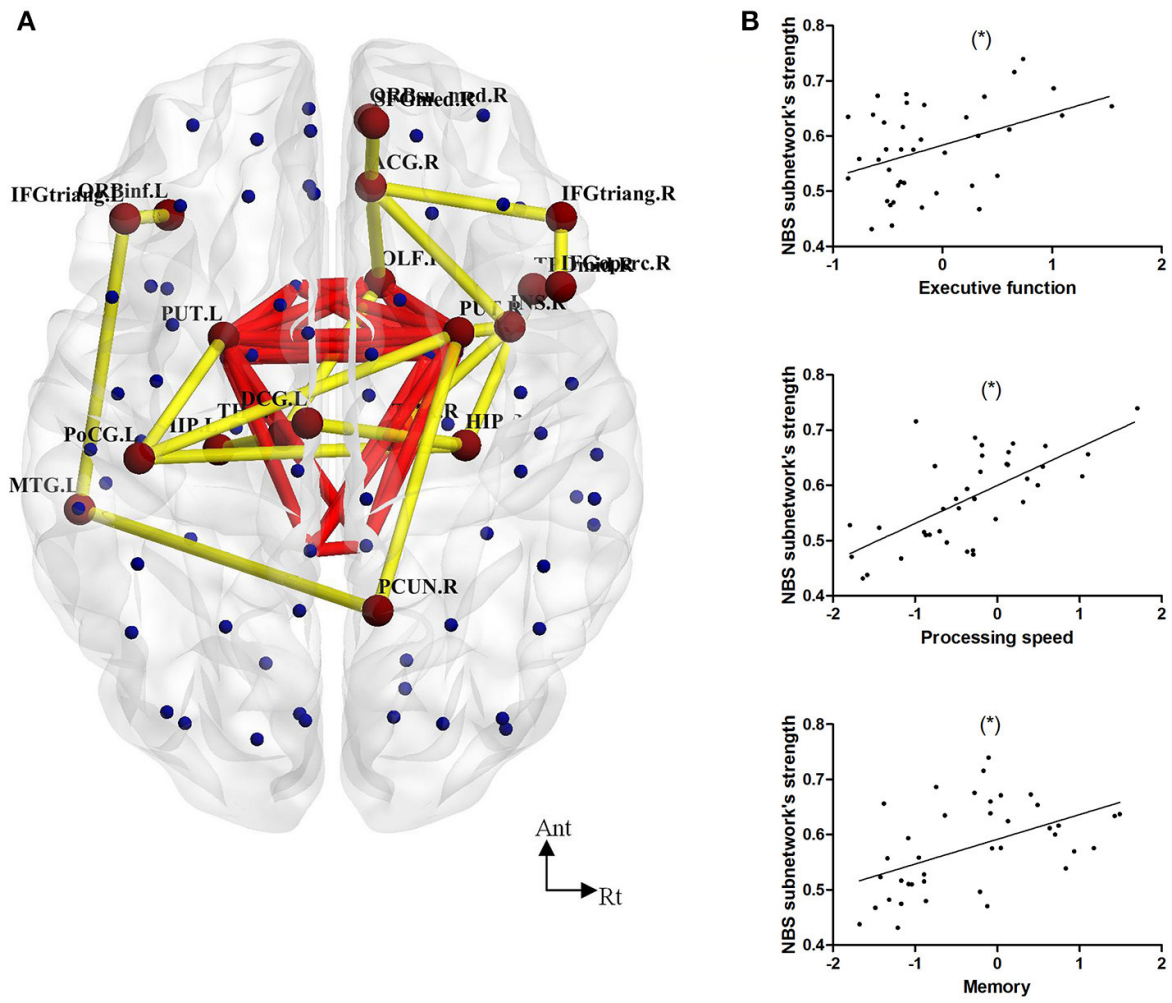
**(A)** NBS analysis showed decreased connections in 20 nodes and 20 edges in LLD-MD compared with the LLD-IM group. Yellow lines represent the edges of the subnetwork connection according to NBS analysis. Red lines represent the rich-club connections. **(B)** The Pearson correlation between NBS subnetwork connective strength and cognitive function in the LLD groups (adjust with HDRS; ^*^P < 0.05/3). The nodes and connections were mapped using BrainNet Viewer software (http://www.nitrc.org/projects/bnv/).

We found a series of different connections among the LLD groups (LLD-MD < LLD-IM, *P* = 0.004) according to NBS analysis. The y included 20 nodes and 20 edges. The nodes in this NBS's subnetwork were primarily composed of some frontal [right opercular part of the inferior frontal gyrus (IFGoperc.R), left triangle part of the inferior frontal gyrus (IFGtriang.L), right triangle part of the inferior frontal gyrus (IFGtriang.R), left orbital part of the inferior frontal gyrus (ORBinf.L)], paralimbic [right olfactory cortex (OLF.R), medial part of the superior frontal (SFGmed.R), right medial orbital part of the superior frontal gyrus (ORBsupmed.R), right insula (INS.R), right anterior cingulate cortex (ACG.R), left middle cingulate cortex (DCG.L), temporal pole of middle temporal gyrus (TPOmid.R)], subcortical[left hippocampus (HIP.L), right hippocampus (HIP.R), left putamen (PUT.L), right putamen (PUT.R), left thalamus (THA.L), right thalamus (THA.R)], parietal [left postcentral gyrus (PoCG.L), right precuneus (PCUN.R)] and the left middle temporal gyrus nodes (MTG.L). The connections were as follows: (IFGoperc.R-IFGtriang.R); (IFGtriang.L-ORBinf.L); (SFGmed.R-ORBsupmed.R); (IFGtriang.R-ACG.R); (OLF.R-ACG.R); (SFGmed.R-ACG.R); (INS.R-ACG.R); (DCG.L-HIP.R); (HIP.R-PoCG.L); (PoCG.L-PUT.L); (OLF.R-PUT.R); (INS.R-PUT.R); (PoCG.L-PUT.R); (PCUN.R-PUT.R); (OLF.R-THA.L); (HIP.L-THA.L); (INS.R-THA.R); (IFGtriang.L-MTG.L); (PCUN.R-MTG.L); (HIP.R-TPOmid.R). In this NBS's subnetwork, 1 edge belonged to a rich-club connection, 6 edges belonged to the feeder connections, and the rest of the 13 edges belonged to the local connections in the previous description (show in Figure 3). Furthermore, the connective strength of this subnetwork was positively correlated with executive function (*r* = 0.424, *P* = 0.008), processing speed (*r* = 0.667, *P* < 0.001) and memory (*r* = 0.490, *P* = 0.002) in LLD patients. No such correlation was found in HC individuals (as shown in Figure 3).

## Discussion

To the best of our knowledge, this is the first time to investigate the rich-club coefficient of the white matter network of LLD patients with different memory states. LLD as one of the most predictable signs of developing AD (Heser et al., 2016), a comprehensive vision of the structural features of this disease, especially those associated with memory deficits, are crucial for understanding the disease process of LLD and the mechanism of AD development. In the present study, our findings in the white matter network change in LLD patients with or without memory deficits demonstrate that (1) impaired rich-club organization, rich-club connective average strength and assortativity was found in 39 LLD patients compared to 30 HC subjects; (2) compared to LLD-IM patients, LLD-MD patients had disruptive feeder and local connections, especially in cognitive control network (CCN) and corticostriatal circuits; and (3) the alterations of global network properties were accompanied by slight cognitive function change, such as processing speed and memory in the current study. Our findings were compatible with the previous study in the white matter network topological features of LLD patients (Bai et al., 2012; Li et al., 2017) to some extent, and extend our understanding in the pathological progression of LLD by describing the rich-club properties of LLD-MD patients.

In the current study, compared to HC individuals, rich-club organization was disrupted in LLD patients, and this phenomenon was independent of memory function. Unlike the other connectome's findings in depression (Bohr et al., 2012), which showed no correlation between depression and global network properties. Our findings suggested that the disease effects of LLD, other than memory deficits, were more prevalent in rich-club regions, which was partly consistent with a previous study about schizophrenia (van den Heuvel et al., 2013) and high-risk subjects for psychosis (Collin et al., 2014; Schmidt et al., 2017). As the central part of brain network, rich-club plays an important role in information integration among different functional modules (van den Heuvel et al., 2013). In major depressive disorder, disruptive functional modules was found and relevant with the feelings of helplessness (Peng et al., 2014). Combined with the established evidence described above, we consider this is the reason for rich-club organization and rich-club connective strength being disrupted in LLD. Striatal connection is crucial in depressive symptom, including suicide and treatment response (Lui et al., 2011; Marchand et al., 2013). Its activity is reduced in depression (Arrondo et al., 2015) and increased accompanied with treatment response (Heller et al., 2013). In the current study, rich-club nodes were including bilateral putamen and pallidum, and rich-club connectivity was decreased in LLD group, indicated that the rich-club connectivity could correlate with the disease state. But we failed to find the correlation between HDRS and rich-club coefficient in the current study. Considering the white matter change can be persisted in a relatively long period, and HDRS can only reflect the current affective state and affected by various of factors. The rich-club coefficient might be consistent with some indicator that can reflect the disease state in a relatively long periods, like the course of disease. Further studies should force on determine the association between the course of disease and rich-club organization, as well as the effects of rich-club organization on network modules in LLD.

Feeder and local connective average strength were decreased in the LLD-MD group compared to the LLD-IM and HC groups in current study. Similar damage pattern was appeared in AD (Daianu et al., 2015). According to our current knowledge, as the center of AD pathological development, amyloid and tau can spread through fibers in a “prion-like” pattern and cause extensive brain damage along white matter fibers (Cohen et al., 2015; Hasegawa et al., 2016; Pandya et al., 2016). This phenomenon is partly consistent with the series of studies from Daianu et al. (2013, 2015), who reported that AD tends to disrupt the non-rich-club regions of the white matter network. Combined with Daianu et al.'s finding, our result indicated that the pathological mechanism of memory deficits in LLD might be similar to those in AD. Previous evidence from clinical data (Rushing et al., 2014; Heser et al., 2016) showed that LLD patients, especially LLD patients with memory deficits, have a higher risk of subsequent AD. Our findings provide further evidence to support the idea that LLD-MD could be a preclinical stage of AD. However, this idea needs further pathological study, including PIB-PET or autopsy, to confirm. In addition, a positive correlation between those subnetwork connective average strength and cognitive function was noted in LLD patients. Combined with stepwise linear regression, we found that executive function was significantly correlated with feeder connective strength and memory was correlated with local connective strength. Processing speed was relevant with rich-club and local connective strength, while local connections seem more important in processing speed than rich-club connections. However, such relevant was lacked in HC group. Our finding further indicated that the essential role of local connections in LLD patient's memory and processing speed. According to the previous neuropsychological study in LLD (Sheline et al., 2006), which demonstrated that processing speed is the primary dysfunction in LLD cognitive deficit and the cause of dysfunction in other cognitive domains. So, it is reasonable to conduct that integrity of the local connections may reflect processing speed, which's disruption might have a negative effect on memory in LLD. However, we cannot determine whether the rich-club disorganization is essential for the effects of local connections in cognitive functions among LLD patient. To answer this question, subjects with disruptive local connections and relatively preserved integrated rich-club organization, such as AD patients, should be included in future studies. What is more, feeder connective strength was correlated with executive function. As we describe above, feeder connections play an essential role in information transfer between rich-club connections and local connections by connecting them together. It is established that dynamic reconfiguration of frontal networks is the foundation of executive function (Braun et al., 2015). Considering the rich-club nodes in this study was involved with amygdala, which is connecting with frontal network through cingulum. So, it is hard to determinate whether the abnormal frontal brain network, which is belonging to feeder subnetwork, or the disruptive feeder subnetwork itself is contributed to executive dysfunction in LLD-MD. And it is worth further study to figure out.

CCN and corticostriatal circuits are comprised of the dorsolateral prefrontal cortex (DLPFC), dorsal anterior cingulate cortex (dACC), posterior parietal cortex, basal ganglia and thalamus, and they are essential in attention and executive function (Tadayonnejad and Ajilore, 2014). According to our NBS analysis, an altered subnetwork connection between LLD-MD and LLD-IM patients comprises the frontal, paralimbic, subcortical and some parietal and temporal regions, which are mainly involved in the right CCN and corticostriatal circuits as well as belonging to feeder and local connections, and the strength of this NBS's subnetwork is correlated with cognitive function in LLD groups. Different from other articles in brain structural change and memory, which show mainly difference in default mode network (Chang et al., 2015; Yin et al., 2016), our finding suggested that the memory deficits in LLD patients is relevant with the disruption of the right CCN and corticostriatal circuits. In the current study, this NBS's subnetwork is mostly belonging to feeder and local connections. It is established that CCN and corticostriatal circuits are involved in cognitive control and emotional behavior (Tadayonnejad and Ajilore, 2014). And cognitive theory showed that disruption in executive dysfunction can impair the act of remembering (Buckner, 2004; Elderkin-Thompson et al., 2006). And cognitive control is in the central part of executive function. Previous studies indicated that memory and cognitive control are relevant at some extent. This effect will enlarge as the subject was distracted by irrelevant events or engage in mutiple-task, and lead to memory deficits (Wais et al., 2010; Richter and Yeung, 2012). Furthermore, depression patient is easy to distract by surrounding environment, especially by negative stimuli (Maalouf et al., 2012). Thus, it is reasonable to conduct that cognitive control as an important part of executive function, its impairment may result in memory deficits, especially in depressive subject. In conclusion, our NBS finding indicated that right CCN and corticostriatal circuit's disruption, which would cause impaired cognitive control according to establish evidence, is correlated with memory deficits in LLD. In our consideration, this result may be caused by the negative effect of cognitive control on recollection, which was enlarged under the disease state of LLD.

Compared to HC, LLD-IM patients had increased local connective average strength. This finding reminds us of the increased fractional anisotropy in the subjects with a genetically high risk for schizophrenia, which indicated there are compensatory effects in white matter for the high genetic risk of schizophrenia (Kim et al., 2012). Similar findings can also be seen in major depressive disorder (Wang et al., 2014) and posttraumatic stress disorder (Li et al., 2016). Therefore, it is reasonable to speculate that an increase in the local connections of LLD-IM patients in the current study could also have a compensatory change against the disease effects of LLD. This change could be the reason LLD-IM patients still maintain memory capacity to some degree.

According to a weight rich-club analysis study among at-risk mental state subjects by Schmidt et al. (2017). The identified rich-club nodes included the dorsal and ventral striatum, the globus pallidus, and the amygdala. Although different from Schmidt's study, who used FreeSurfer software to parcellate the brain into 82 cortical and subcortical regions, we found nearly the same rich-club nodes that were mentioned above by using FSL to parcellate the brain into 90 cortical and subcortical regions, except for the bilateral olfactory was found in ours. Our finding was different from other rich-club study findings (van den Heuvel and Sporns, 2011; Daianu et al., 2015), and the established rich-club nodes in these studies included the superior parietal, precuneus, superior frontal cortex, putamen, hippocampus, and thalamus. Similar to the views of Schmidt et al. different identification of richness factors and weight normalization methods could be the most likely explanations for this discrepancy.

Disruption in the global network properties in LLD patients has been shown in previous articles (Bai et al., 2012; Li et al., 2015), but none of them were focused on the effects of cognitive function in LLD patients, which was relevant to the integrity of white matter in LLD (Sexton et al., 2012; Li et al., 2017). Similar to previous articles (Bai et al., 2012; Gong and He, 2015; Li et al., 2017), global network properties, such as the shortest path length, connective strength, efficiency and fault tolerant efficiency, were disrupted in LLD-MD patients compared to LLD-IM patients and HC individuals, while groups did not differ in the clustering coefficient and small world properties in our study. Density of network was significantly decreased in LLD-IM compared with LLD-MD and HC groups. The shortest path length, connective strength and efficiency are related to the inter regional effective integrity in the network and the ability for information transmission between remote regions. In the present study, impairment of these properties in LLD-MD patients indicated the relative inefficiency of connection. Additionally, compared to LLD-IM patients and HC individuals, impaired fault tolerant efficiency reflected the increasing vulnerability of the network in LLD-MD patients. Similar to recent findings in the LLD connectome (Li et al., 2017), global network properties had a slightly positive correlation with processing speed and memory in the current study, which showed that impaired global network properties can reflect cognitive function to some extent. Furthermore, density of network was significantly decreased in LLD-IM compared with LLD-MD and HC groups. Despite the density of LLD-IM's network was lower than LLD-MD's, the connective strength of LLD-IM was still stronger than LLD-MD. Indicated that the LLD-IM have more effective connections than LLD-MD, while the density increase in LLD-MD may due to the compensatory effects of the disease state of memory deficit. But this conduction needs further research to verify.

A network with positive assortativity tends to have central nodes with high interconnection (Newman, 2002), which is similar to the rich-club coefficient to some extent. In the current study, positive assortativity was found in all participants. In addition, LLD patients have lower assortativity than HC, which was consistent with our rich-club organization findings. These results not only support the existence of rich-club organization findings in the white matter network, but also indicate that the disease effects of LLD might primarily disrupt interconnection among central nodes. Moreover, assortativity was positively correlated with processing speed as other global network measures in this study. Not only fault tolerant efficiency but also higher assortativity was represented for greater resilient to target node insults in the network (Cisler et al., 2016). Accompanied by the other global network property findings mentioned above, our results suggested that disrupting the integrity and vulnerability of the brain network can be the hallmark of information transfer impairment during cognitive processes in LLD patients.

There are some limitations to this study besides the limited sample size. (1) Different parcellation resolutions might lead to different findings on graph analytical findings in the human brain (Fornito et al., 2010). However, to avoid the “fiber crossing problem” in deterministic tractography (Bai et al., 2012), probabilistic tractography was used to map the brain fiber connectivity in the current study, which is too computationally intensive to use high resolution analysis to verify the findings in an AAL90 atlas. Because of the limitations of a large data-processing capacity, we could not use a high resolution analysis in this study. (2) In the current study, we did not group the LLD patients into episodes or remission stage by HRSD. Although the effects of depressive episodes on cognitive function have been proven in an earlier study, which called this phenomenon “depressive pseudodementia” (Bulbena and Berrios, 1986), those effects did not influence our findings. HRSD is a psychological test to evaluate a subject's emotional state for the prior month, which has limited influence on white matter changes. Additionally, subjects who were unable to cooperate with the neuropsychological battery were excluded and subjects who were recruited were confirmed by their psychiatrists to be in a relapse stage. What is more, HDRS was adjusted in the statistics involves in cognitive ability. We consider the procedures above to be sufficient for guaranteeing the minimum effects of a depressive episode on cognitive function. Furthermore, no significant differences in HRSD were found between the LLD groups, and there was no association between HRSD and cognitive function in the current study. This finding is similar to the study of McCutcheon, S.T, who reported that no correlation was found between Geriatric Depression Scale (GDS) and cognitive function, while GDS is a scale similar to HRSD, except for self-rating and a specific design for elderly subjects (McCutcheon et al., 2016). Therefore, there is very little influence of HRSD on our findings and this influence as a covariance was excluded from this study.

In summary, the current study extends our understanding of the disease progression of LLD by demonstrating that the disease effects of LLD were mostly prevalent in the rich-club organization among elderly individuals. In addition, breakdown of feeder and local connections, especially right CCN and corticostriatal circuits, played an essential role in memory deficits among LLD patients, which may be the underlying mechanism of AD development. Additionally, our findings also demonstrated that impaired global network properties could reflect cognitive dysfunction in LLD patients to some extent. Further studies should aim to reveal the interaction between rich-club disorganization and non-rich-club connections on cognitive function in the human brain.

## Author contributions

NM and YN designed experiments; NM, XZ, BC, QP, ZW, WZ, and CO carried out experiments; NM wrote the manuscript and analyzed experimental results.

### Conflict of interest statement

The authors declare that the research was conducted in the absence of any commercial or financial relationships that could be construed as a potential conflict of interest.
