# Supplementary material for: Weight Rich-Club Analysis in the White Matter Network of Late-Life Depression with Memory Deficits
Source: Front Aging Neurosci. 2017 Aug 23;9:279. doi: 10.3389/fnagi.2017.00279 (PMC5572942; doi:10.3389/fnagi.2017.00279)
Supplement: Supplementary file 1 [file Table1.DOCX]

**Supplemental material**

Supplemental table 1. Pearson correlations between global network properties, HRSD and cognitive function.(N=69)

|  |  | Cp | Lp | S | E_glob | Eloc | Assortativity | Density |
| --- | --- | --- | --- | --- | --- | --- | --- | --- |
| Executive function | r | -0.004 | -0.158 | 0.150 | 0.151 | 0.157 | 0.005 | -0.078 |
|  | *P* value | 0.976 | 0.198 | 0.221 | 0.218 | 0.201 | 0.969 | 0.528 |
| Processing speed | r | -0.019 | -0.283^*^ | 0.328^**^ | 0.287^*^ | 0.302^**^ | 0.363^**^ | 0.214 |
|  | *P* value | 0.875 | 0.019 | 0.006 | 0.018 | 0.012 | 0.002 | 0.080 |
| Memory | r | -0.042 | -0.335^**^ | 0.347^**^ | 0.332^**^ | 0.321^**^ | 0.247^*^ | 0.109 |
|  | *P* value | 0.736 | 0.005 | 0.004 | 0.006 | 0.008 | 0.042 | 0.376 |

Adjusted for HDRS.^*^*P*<0.05;^**^ *P*<0.05/3

Cp: clustering coefficient; Lp; shortest path length; S: strength; E_glob: efficiency; Eloc: fault tolerant efficiency; r: assortativity.

Supplemental table 2. Pearson correlation between cognitive function and rich-club properties in LLDs and HC.

|  |  |  | Rich-club coefficient (r=2/15) | area under curve of rich-club coefficient | Rich-club connective average strength | feeder connective average strength | local connective average strength | HRDS^#^ |
| --- | --- | --- | --- | --- | --- | --- | --- | --- |
| Executive function | LLDs (N=39) | r | -0.099 | -0.114 | 0.165 | 0.416 | 0.361 | -0.066 |
|  |  | *P* value | 0.556 | 0.497 | 0.323 | 0.009^**^ | 0.026^*^ | 0.688 |
|  | HC (N=30) | r | 0.112 | 0.020 | -0.125 | -0.208 | -0.116 | 0.110 |
|  |  | *P* value | 0.564 | 0.916 | 0.518 | 0.278 | 0.548 | 0.563 |
| Processing speed | LLDs (N=39) | r | -0.188 | -0.020 | 0.369 | 0.411 | 0.555 | 0.129 |
|  |  | *P* value) | 0.259 | 0.905 | 0.023^*^ | 0.010^**^ | <0.001^**^ | 0.434 |
|  | HC (N=30) | r | -0.132 | -0.039 | -0.261 | -0.274 | -0.175 | 0.047 |
|  |  | *P* value | 0.496 | 0.840 | 0.171 | 0.150 | 0.363 | 0.807 |
| Memory | LLDs (N=39) | r | 0.164 | 0.153 | 0.330 | 0.295 | 0.432 | 0.029 |
|  |  | *P* value | 0.325 | 0.361 | 0.043^*^ | 0.073 | 0.004^**^ | 0.863 |
|  | HC (N=30) | r | 0.007 | 0.007 | -0.006 | 0.047 | -0.082 | 0.003 |
|  |  | *P* value | 0.971 | 0.971 | 0.974 | 0.808 | 0.672 | 0.987 |

Adjusted for HDRS.^*^*P*<0.05;^**^ *P*<0.05/3;

^#^ Not adjusted for HDRS
